# *In Vitro* Cytotoxicity and Mineralization Potential of an Endodontic Bioceramic Material

**DOI:** 10.1055/s-0042-1750778

**Published:** 2022-10-03

**Authors:** Soumya Sheela, Mohannad Nassar, Fatma M. AlGhalban, Mehmet O. Gorduysus

**Affiliations:** 1 Dental Biomaterials and Biomimetics Research Group, Research Institute for Medical and Health Sciences, University of Sharjah, Sharjah, United Arab Emirates; 2 Department of Preventive and Restorative Dentistry, College of Dental Medicine, University of Sharjah, Sharjah, United Arab Emirates

**Keywords:** bioceramics, osteoblasts, mineralization, total fill BC, AH Plus

## Abstract

**Objective** The interest in bioceramic materials has been steadily growing for different applications in endodontics. With the continued introduction of new bioceramic-based materials into the market, it is of great importance to assess the biocompatibility before providing recommendations on their clinical use. This study evaluated the *in vitro* cytotoxicity and mineralization potential of two consistencies of unset premixed bioceramic material (TotalFill BC RRM putty and TotalFill BC sealer) compared with an epoxy resin-based sealer (AH Plus) on osteoblast cells.

**Materials and Methods** Overall, 100% extracts were obtained by weighing 0.1 g of each material in 1 mL of cell culture media. Primary human osteoblast (HOB) cells ( *n* = 4) were treated with different concentrations (100, 50, 25, 12.50, and 6.25%) of each extract. XTT assay and Alizarin Red S staining were used to evaluate the cytotoxic effect and the biomineralization potential, respectively.

**Statistical Analysis** Data were analyzed by one-way analysis of variance followed by Tukey's post hoc tests.

**Results** The cytotoxicity assay after 24 h treatment showed that all materials at high concentrations of the extract (100 and 50%) were toxic to HOB ( *p* < 0.001). On the contrary to TotalFill BC RRM Putty, AH Plus and TotalFill BC sealer were toxic at 25% concentration. However, at 12.5% concentration and lower, all materials were nontoxic. The mineralization potential analyzed after 7 and 14 days showed that TotalFill BC material–treated cells could deposit mineralized nodules in the normal and osteogenic medium unlike AH plus-treated cells.

**Conclusion** At low concentrations, TotalFill BC materials showed higher biocompatibility to HOB cells than AH Plus, enhanced the viability of the cells, maintained their typical morphology, and induced the formation of mineralized nodules. Despite the encouraging data, clinical trials are needed to identify the effect of this material on the long-term outcome of endodontic treatment.

## Introduction

Despite gutta-percha being the core and primary obturating material in root canal treatment, sealers are still being used to fill the gaps between the core material and the root canal walls and to lubricate the space during the obturation procedure. This helps achieve a three-dimensional fluid-tight seal, thus preventing leakage and bacterial growth. All while being biocompatible to vital structures and without impairing healing at the periapical tissue surrounding the tooth being treated. 1 2 3 Schroder introduced AH26 sealer as a root canal filling material. AH26 mainly contains bismuth oxide and hexamethylenetetramine as powder and bisphenol A diglycidyl ether as a resin. 4 The other formulation of AH26 and the most used one is called AH Plus (Dentsply DeTrey, Konstanz, Germany) which is composed of epoxy paste, such as diepoxy; calcium tungstate; zirconium oxide; aerosol; and dye amine paste such as 1-adamantane amine, N'dibenzyl-5 oxanonandiamine-1,9, TCD-diamine, calcium tungstate, zirconium oxide, aerosol, and silicone oil. 5 The AH Plus allows a better mix, has more radiopacity, shorter setting time, lower solubility, better flow, and, more importantly, does not release formaldehyde on setting. 6 It also does not require the use of dentin adhesive. 7 Currently, AH Plus is the most studied sealer over the past 20 years and is considered a gold standard compared with newer types of sealers. Despite the several advantages offered by AH plus, a recent review of the literature has reported that most of the studies agree on the cytotoxic effect of AH Plus, especially in freshly mixed conditions. 8

Tricalcium silicate materials, also known as bioceramic cement, are viewed as essential bioactive materials in different fields of dentistry. The primary applications of these materials in endodontics are vital pulp therapies, perforation repair, and root-end filling. 9 A novel category of sealers based on tricalcium silicate technology has been developed. Despite their higher cost, they are gaining much attention from researchers and clinicians alike, driven by their assumed biocompatibility, ability to deposit apatite-like crystals, and ease of use. 10 Despite the notion that tricalcium silicate-based sealers display better biocompatibility than resin-based sealers, there is a lack of consensus in the literature regarding this matter. 11 12 13 14 The different results seen in the literature are attributed to several factors, such as the condition and time of material setting, sealer concentration, and exposure time. 8 In the past 15 years, quite a few companies have stepped up to the plate and produced their tricalcium silicate-based materials for root canal treatment application purposes. Thus, combined with the increased demand, there is a continued need to adequately evaluate these products before their introduction into the market for clinical use. 15 16 A well-known and probably the current dominant representative of the new generation of bioceramic materials is EndoSequence BC (Brasseler, Savannah, Georgia, United States) that has found its way into the European dental market as TotalFill BC (FKG Dentaire, La Chaux-de-Fonds, Switzerland) 17 which is composed of zirconium oxide, calcium silicates, calcium phosphate monobasic, calcium hydroxide, filler, and thickening agents. 5

Several factors can negatively impact the outcome of root canal treatment, and one of them is sealer extrusion in its fresh or unset condition into the periapical tissues. 18 19 Thus, the purpose of this study was to evaluate the *in vitro* cytotoxicity and mineralization potential of two consistencies of unset premixed bioceramic material (TotalFill BC) compared with an epoxy resin-based sealer (AH Plus) on human osteoblast (HOB) cells. The null hypotheses tested were no differences in the cytotoxicity and mineralization potential within the tested materials or the used dilutions.

## Materials and Methods

The test materials, product names, manufacturers, and composition are listed in Table 1 . The materials were handled according to the manufacturers' instructions. The tested materials were mixed in DMEM/F-12-cell culture media (Gibco; Thermo Fisher Scientific, Inc., Waltham, Massachusetts, United States) supplemented with 10% fetal bovine serum (FBS) and 1% penicillin-streptomycin (Sigma-Aldrich, St. Louis, Missouri, United States) in such a way to obtain a concentration of 0.1 g/mL following the International Organization for Standardization (ISO) 10933–12 standards. The 100% extract was obtained by weighing 0.1 g of the test samples in 1 mL of the culture medium and then incubated for 24 at 37°C in a humidified atmosphere of 5% CO _2_ . All the extracts were filtered using a 0.22-µm syringe filter before treating the cells. Different concentrations of the extract of the test samples were prepared, 100, 50, 25, 12.50, and 6.25% for treating HOB cells.

**Table 1:** Manufacturers and chemical compositions of tested materials

Material	Components	Lot number	Ingredients
AH Plus (Detrey Dentsply, Konstanz, Germany)	Paste A Paste B	1903001256 1903001255	Epoxy resin, calcium tungstate, zirconium oxide, aerosol, iron oxide adamantane amine, N,N-dibenzoyl-5-oxanonane-diamine-1,9, TCD-diamine, calcium tungstate, zirconium oxide, silicone oil, aerosil
TotalFill BC RRM Putty (FKG Dentaire SA, Switzerland)	Putty jar	1902BPP	Zirconium oxide, calcium silicates, calcium phosphate monobasic, calcium hydroxide, filler and thickening agents
TotalFill BC Sealer (FKG Dentaire SA, Switzerland)	Preloaded syringe	19003SP	Zirconium oxide, calcium silicates, calcium phosphate monobasic, calcium hydroxide, filler and thickening agents

### Human Osteoblast Cell Line Culture

HOB cells were obtained from AddexBio (AddexBio P0004010, San Diego, California, United States) and maintained in DMEM/F-12 culture medium supplemented with 10% fetal bovine serum and 1% penicillin-streptomycin (Sigma-Aldrich). The cells were maintained at 37°C in 95% O _2_ and 5% CO _2_ humidified atmosphere.

### Cell Viability Assay of Osteoblast

An XTT (Cell Proliferation Kit II [XTT], Sigma-Aldrich, St. Louis, Missouri, United States) assay was performed to assess the toxic effect of different bioceramic sealers on the viability of HOB cells. The test on extract method was followed as per the ISO standards. Briefly, HOB cells were seeded onto 96-well cell culture-treated plates at a density of 10 ^4^ cells/well ( *n* = 4) in a final volume of 100 μL of complete DMEM/F-12 culture medium. The cells were kept in the incubator for 24 h to get a monolayer culture. After 24 h, the cell culture medium was replaced with extract medium (100, 50, 25, 12.50, and 6.25%) before the cytotoxicity evaluation was performed. Untreated cells were used as controls. After an incubation time of 24 h, 50 μL of the XTT labeling mixture was added to each well following the manufacturers' instructions and was kept for 4 h. The concentration of orange soluble formazan product formed was measured using plate reader (Synergy H1 microplate reader, Biotek Instruments, United States) at 450 and 630nm wavelengths. Four replicates of each concentration were performed in each test. All assays were repeated three times to ensure reproducibility. Cell viability was calculated as the percentage of the control group. Data were analyzed by one-way analysis of variance (ANOVA) followed by Tukey's post hoc tests. The level of significance was set at 0.05. GraphPad Prism version 5.03 software (GraphPad Software, Inc., San Diego, California, United States) was used for statistical analyses in this study.

### Calcium Mineral Deposition by Alizarin Red S Assay

For analyzing the mineralization ability of the different bioceramic sealers, HOB cells at a density of 50,000/well ( *n* = 4) were seeded onto six-well plates and maintained in DMEM/F-12 media (NM). On confluency, the media was changed to osteogenic media (OM) (10 ^-8^ M dexamethasone, 10 mM beta glycerophosphate, and 50µg/mL ascorbic acid). The extract of the test materials (25, 12.5, and 6.25%) were added to HOB cells and were cultured for 14 days in either osteogenic or normal media. Media change was performed once in 3 days. After 7 and 14 days, the plates were taken and fixed using 4% paraformaldehyde for 20 min at room temperature. Subsequently, the plates were washed with distilled water and stained with 40mM Alizarin Red S solution (Sigma-Aldrich; pH = 4.1) for 20 to 30 mins at room temperature and the stained calcium deposits were captured using light inverted microscope (Olympus IX53, Olympus Corporation, Tokyo, Japan).

## Results

### Cell Viability Assay

**Fig. 1:**
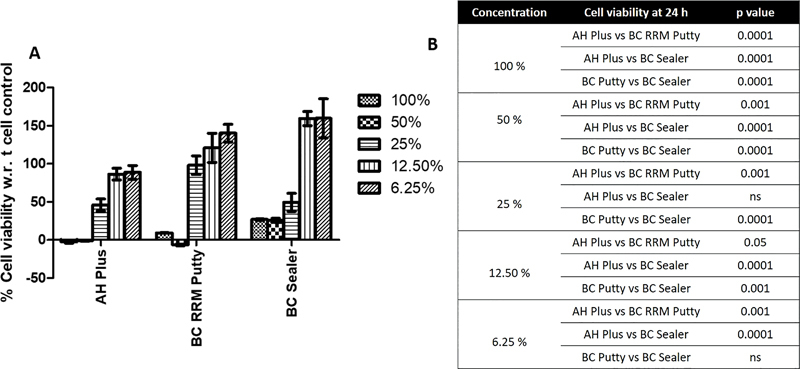
( **A** ) Human osteoblast cells viability after 24 h exposure to different concentrations (100, 50, 25, 12.5, or 6.25%) of extracts of the tested materials: AH plus, TotalFill BC RRM Putty, and TotalFill BC Sealer. Cell viability was determined by using XTT assay and cell viability was calculated as the percentage of the control group. ( **B** ) The statistical significance between the tested groups. Data were analyzed using one-way analysis of variance (ANOVA).

The effects on osteoblast cells after 24 h exposure to extracts of AH Plus and TotalFill BC RRM Putty and BC Sealer are presented in Fig. 1 . At high concentrations (100 and 50%), all tested materials were toxic on the used cells ( *p* < 0.0001). TotalFill BC Sealer and AH Plus were toxic at even 25% concentration of the extract as they demonstrated cell viability of around 50% when compared with 100% cell viability exhibited by TotalFill BC RRM Putty ( *p* < 0.001). At 12.5% concentration and lower, all materials were found to be non-toxic; however, a significant increase in cell viability was demonstrated by TotaFill BC RRM Putty and BC Sealer when compared with AH Plus ( *p* < 0.05). Between TotalFill BC RRM Putty and BC Sealer, the latter showed a significant increase in cell viability at 12.5% of extract concentration ( *p* < 0.001). When 6.25% of the extract concentration was used, TotalFill BC RRM Putty and BC sealer showed a significant increase in cell viability when compared with AH Plus ( *p* < 0.001). However, the difference between TotaFill BC Sealer and BC RRM Putty was non-significant ( *p* > 0.05)

**Fig. 2:**
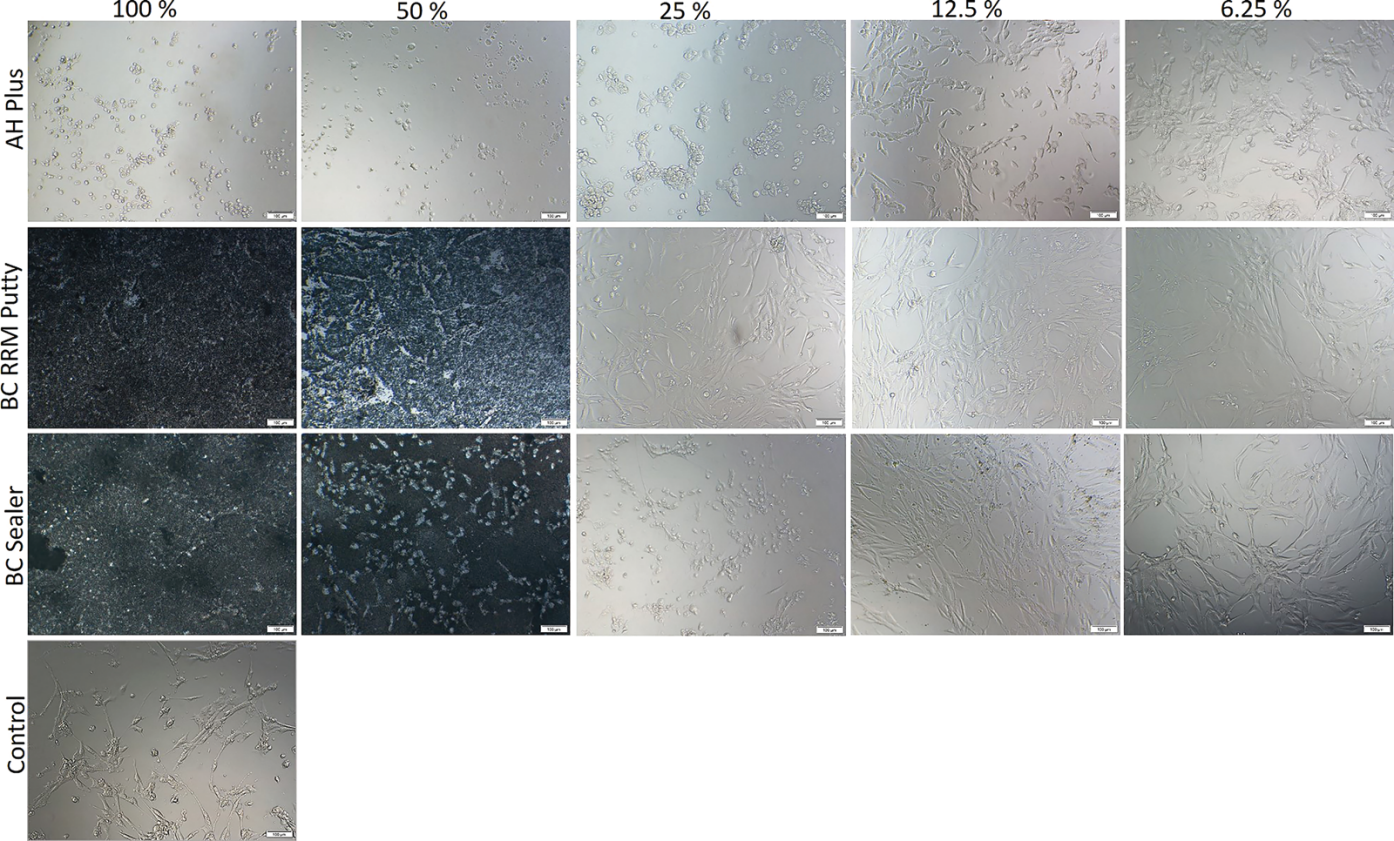
Representative phase-contrast microscopy images of the morphologic changes of human osteoblast cells after 24 h exposure to different concentrations (100, 50, 25, 12.5, or 6.25%) of extracts of the tested materials: AH plus, TotalFill BC RRM Putty, and TotalFill BC Sealer.

The morphology of the HOB cells ( Fig. 2 ) cultured with 100, 50, or 25% of the extract concentrations of AH Plus showed a rounded morphology compared with the normal fibroblastic appearance of the control HOB cells. At 12.5% or lower concentrations of the extract, the cells exhibited the regular fibroblastic morphology as that of the control HOB cells. For the TotalFill BC RRM Putty and BC Sealer groups at 100 and 50% of the extract, the morphology of the cells was disrupted, and the surface was covered with what looked like cellular debris. However, for the TotaFill BC RRM Putty group as confirmed with the viability results mentioned above, the cytotoxicity effect diminished at 25% or lower of the extract concentration, and the cells retained their typical morphology. For the TotalFill BC Sealer group, 25% concentration resulted in a rounded morphology and wide intercellular spaces, while 12.5 and 6.25% concentrations maintained the normal morphology of the cells.

### Effect of Sealers on Osteoblast Mineralization

**Fig. 3:**
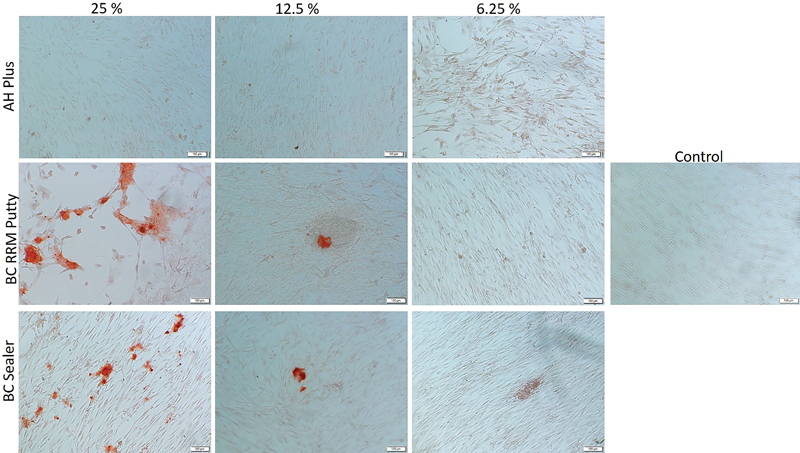
The mineralization of human osteoblast cells treated with different concentrations of the extracts for 7 days in normal medium. The orange-colored calcium deposits stained by Alizarin Red S dye is visible in cells treated with TotalFill BC RRM Putty and BC sealer (25 and 12.5%). AH plus treated cells and the control cells did not show signs of mineralized deposits.

**Fig. 4:**
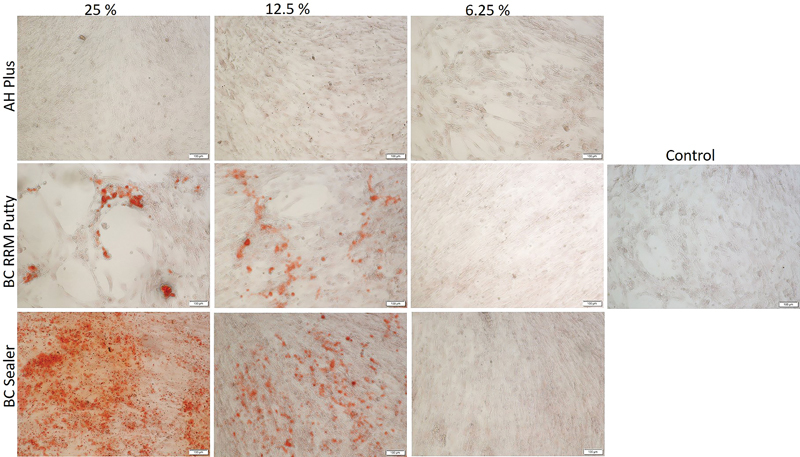
The mineralization of human osteoblast cells treated with different concentrations of the extracts for 14 days in normal medium. The orange-colored calcium deposits stained by Alizarin Red S dye is clearly visible in cells treated with TotalFill BC RRM Putty and BC sealer (25 and 12.5%). AH plus treated cells and the control cells did not show signs of mineralized deposits.

To determine the potential role of the tested materials in mineralization, alizarin Red S staining was performed. The cells were grown in the materials' extracts in either a normal cell culture medium or in a medium supplemented with osteogenic factors. The 25 and 12.5% extracts of TotaFill BC RRM Putty and BC Sealer promoted biomineralization even in the normal cell culture medium. The cells treated with TotaFill BC RRM Putty and BC Sealer extract cultured in the normal medium at 7 and 14 days showed mineral deposition which appeared orange-red after Alizarin Red S staining ( Figs. 3 and 4 ). More mineralized nodules in the normal medium could be observed on day 14 compared with day 7 in TotaFill BC RRM Putty and BC Sealer treated cells. In the normal medium, neither AH Plus-treated cells nor the control cells showed signs of mineralization.

**Fig. 5:**
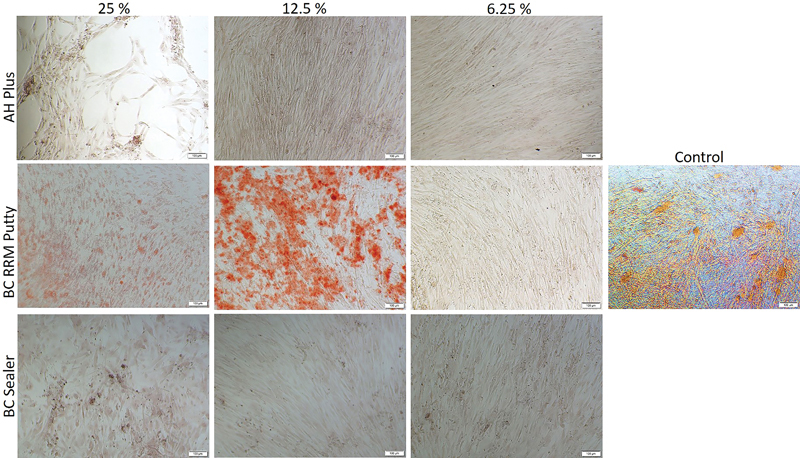
The mineralization of human osteoblast cells treated with different concentrations of the extracts for 7 days in osteogenic medium. The orange colored calcium deposits stained by Alizarin Red S is visible in cells treated with TotalFill BC RRM Putty (12.5%), TotalFill BC RRM Putty (25%), and TotalFill BC Sealer (25%), in a descending order. Control cells showed signs of mineralization; however, AH plus cells lacked the presence of mineralized nodules.

**Fig. 6:**
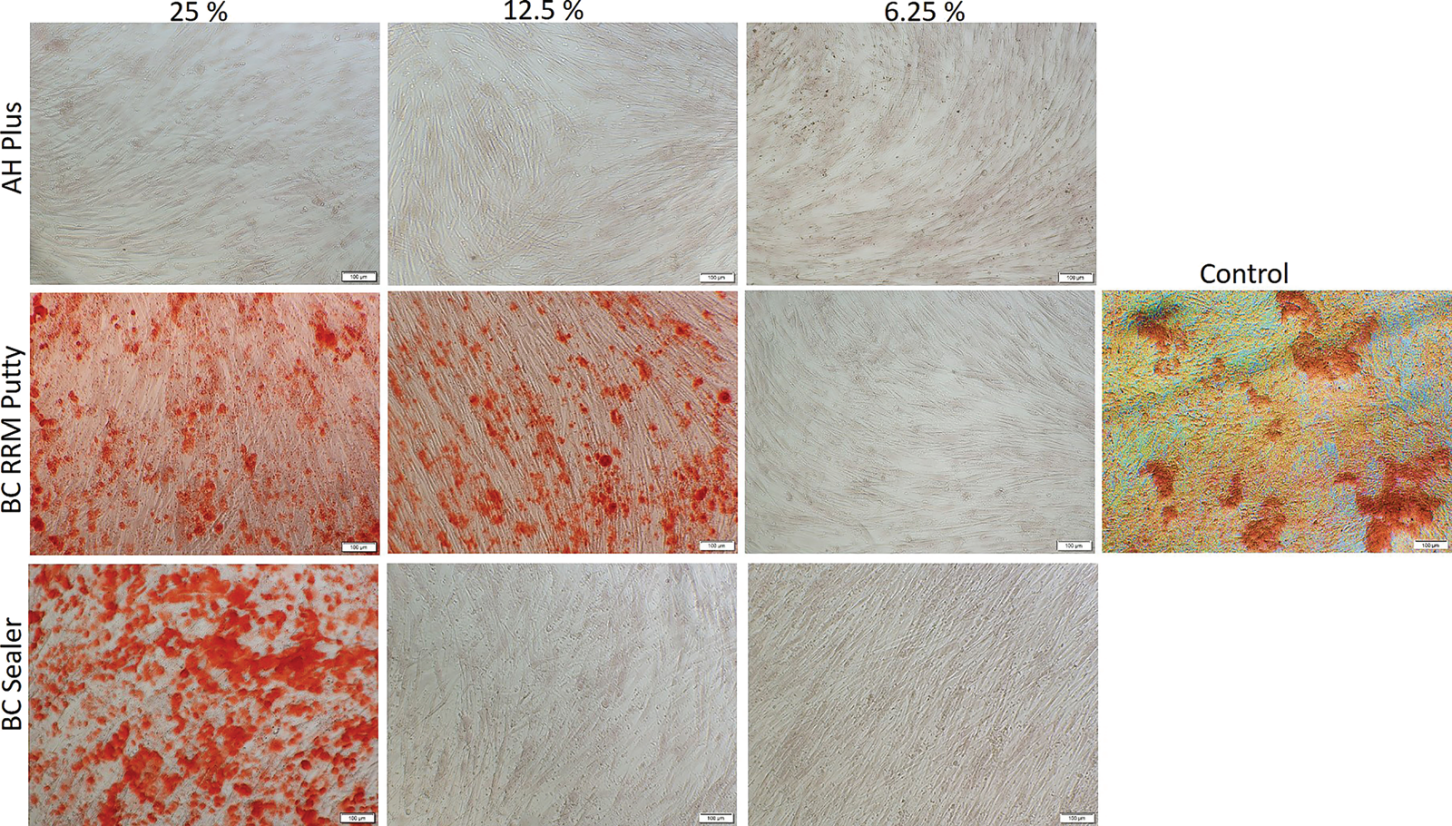
The mineralization of human osteoblast cells treated with different concentrations of the extracts for 14 days in osteogenic medium. The orange colored calcium deposits stained by Alizarin Red S is visible in cells treated with TotalFill BC Sealer (25%), TotalFill BC RRM Putty (12.5%), TotalFill BC RRM Putty (25%), in a descending order. Control cells showed signs of mineralization; however, AH plus cells lacked the presence of mineralized nodules.

Cells treated with TotalFill BC RRM Putty and BC Sealer extracts in the osteogenic medium exhibited mineralized nodules at 7 and 14 days ( Figs. 5 and 6 ). At day 7 in the osteogenic medium, orangish-red precipitate could be mainly seen in TotalFill BC RRM Putty-treated cells (25 and 12.5%) and control cells. However, no deposits were seen on AH Plus and TotalFill BC Sealer extract–treated cell cultures at day 7 in the osteogenic medium. At day 14 in the osteogenic medium, increased mineralized nodules deposition could be seen on TotalFill BC RRM Putty– (25 and 12.5%) and BC Sealer (25%)–treated cells. The control cells showed mineral deposition which could be visualized as orange-red coloration. AH plus-treated cells did not show signs of mineralization at day 14 in the osteogenic medium.

## Discussion

Sealer is still considered a crucial material for use in root canal treatment. The choice of an endodontic sealer should consider its biocompatibility on osteoblast cells due to the potential of extrusion of the sealer in its unset condition into the periapical tissue. Furthermore, it is crucial to sufficiently evaluate new materials that found their way into the dental market before recommending their clinical use. TotalFill BC, with its two different consistencies, is becoming a popular material and is being recommended for several applications in endodontics. This study evaluated the effects of different dilutions of TotalFill BC materials and AH Plus sealer on the viability and mineralization potential of human osteoblasts. The present study results have shown that under unset conditions, the type of material and its dilution significantly affected the viability, mineralization potential, and morphology of human osteoblast cells. These results require the rejection of the null hypotheses.

AH plus has a long track of clinical use, and it is extensively studied in the literature. The present study results are consistent with previous findings where AH Plus was reported to be cytotoxic in its fresh condition even at low dilutions, and its effect is concentration dependent. 13 There are several speculations on the factors that make AH plus toxic to vital tissues when freshly prepared. First, despite the claim that AH Plus is formaldehyde free, it still releases formaldehyde in minimal concentrations. 20 21 Formaldehyde is considered a by-product of the reaction of epoxy resin with the amines. 22 Ho et al reported that formaldehyde has remarkable toxicity on periapical tissues, and even at low concentrations, it can quickly reach a toxic level to human osteoblast cells. Glutathione depletion within the cells is one of the primary mechanisms by which this toxic volatile agent negatively impacts the viability of osteoblast cells. 23 Cohen et al reported that the literature is not clear whether a single or multiple substances are accountable for the toxicity of AH Plus. However, other components are implicated in the toxicity in addition to formaldehyde, namely, amines and epoxy resin. 22 24 The latter is considered the mutagenic part of paste A of AH plus. 25 The involvement of several compounds in the toxicity might explain the comparable cellular toxicities of AH Plus to its predecessor AH 26. 22 25 Osteoblast cells are necessary for the healing of the periapical tissue; thus, it is crucial to assess the effect of sealers on the mineralization ability of these cells. In our study, cells treated with AH Plus showed a lack of formation of mineralized nodules at all exposure times in conventional culture medium and osteogenic medium. Similar to previous findings, 26 the osteoblast cells in the present study lost their polygonal appearance and became retracted and spherical which are signs of cellular degeneration. This can be explained by the effect of AH Plus in initiating a moderate to a severe reaction that results in a significant inflammatory response and bone resorption 27 and its adverse effect on the alkaline phosphatase activity activity of osteoblast cells 28 29 which is a reflection of the mineralization ability of the cells. 30 Indeed, AH Plus is known to release negligible amounts of calcium ions that are needed for the mineralization process of the cells. 31 Overall, these reactions to AH Plus were deemed to have a significant role in the progression of periapical bone destruction. 28

In the TotalFill BC groups, the higher concentrations had a negative impact on cells' viability. The exact mechanism of this type of material that causes cell damage is not clear. However, the high alkaline nature of the unset material might have contributed to the observed lower cell viability. 32 TotalFill BC material, also known as Endosequence BC, has been reported to release a significant amount of hydroxyl ions which elevate the pH of the environment. 33 Despite the beneficial effect of high pH for an enhanced antimicrobial effect of the material, it may also damage the DNA and denaturing proteins of other types of cells. 34 However, at lower concentrations, TotalFill BC exhibited a positive impact on the viability of the cells. The cells also retained their normal spindle-like morphology and produced mineralized nodules at both media at certain concentrations of the extracts. TotalFill BC material contains a considerable amount of calcium silicate which gives rise to calcium hydroxide on hydration, further dissociating into calcium ions in higher amounts than other bioactive materials. 33 Not only are calcium ions essential for the differentiation of the osteoblast cells, but they are also salient for the proper mineralization process and generation of calcium nodules. 13 35 The high amounts of calcium released by this material are speculated to promote periapical healing. 33 The presence of zirconium oxide in TotalFill BC material might also contribute to a favorable outcome compared with other types of oxides such as barium oxide. 36 37 In the present study, the slight differences in the results between TotalFill BC RRM Putty and BC Sealer are probably attributed to different solubilities, as the sealer is made in a less-viscous consistency; however, both share the same composition. 38 With the expanding demand for bioceramic materials in different applications in endodontics, especially as a key obturating material, 39 further research in the form of clinical trials is warranted to understand their effects on the outcome of treatment at their desired applications.

## Conclusion

The limitations of this study include but are not limited to the use of in vitro experimental conditions which contribute to only limited answers to more complex problems. Within these limitations, TotalFill BC materials had a concentration-dependent effect on the viability of human osteoblast cells.

At low concentrations, TotalFill BC materials showed higher biocompatibility to human osteoblast cells than AH Plus, enhanced the viability of the cells, maintained their typical morphology, and induced the formation of mineralized nodules. While these findings are of interest, their clinical relevance remains to be determined.

## References

[1] Endodontic Sealing Material, ANSI/ADA Specification no. 57. Chicago, Ill, USA: American National Standards/American Dental Association;2000 https://webstore.ansi.org/preview-pages/ADA/preview_ANSI+ADA+Specification+No.+57-2000.pdf

[2] Standard ANDAS Endodontic sealing materials Apollonia (Sydney) [Internet]. 2000 32 04 34, 36-”38, 40–42”

[3] International Standards Organization ISO 6876:2012. Dentistry–root canal sealing materialsAccessed May 9, 2022 at:https://www.iso.org/standard/45117.html

[4] Azar N G Heidari M Bahrami Z S Shokri F *In vitro* cytotoxicity of a new epoxy resin root canal sealer J Endod 2000 26 08 462 465 11199780 10.1097/00004770-200008000-00008

[5] Rodríguez-Lozano F J López-García S García-Bernal D Chemical composition and bioactivity potential of the new Endosequence BC Sealer formulation HiFlow Int Endod J 2020 53 09 1216 1228 32412113 10.1111/iej.13327

[6] Himel V T McSpadden J T Goodis H E Instruments, materials, and devices St Louis, MO Mosby Elsevier 2005 233 289

[7] Schwartz R S Adhesive dentistry and endodontics. Part 2: bonding in the root canal system-the promise and the problems: a review J Endod 2006 32 12 1125 1134 17174666 10.1016/j.joen.2006.08.003

[8] Fonseca D A Paula A B Marto C M Biocompatibility of root canal sealers: a systematic review of in vitro and in vivo studies Materials (Basel) 2019 12 24 1 34 10.3390/ma12244113 PMC6947586 31818038

[9] Primus C M Tay F R Niu L N Bioactive tri/dicalcium silicate cements for treatment of pulpal and periapical tissues Acta Biomater 2019 96 35 54 31146033 10.1016/j.actbio.2019.05.050 PMC6717675

[10] Komabayashi T Colmenar D Cvach N Bhat A Primus C Imai Y Comprehensive review of current endodontic sealers Dent Mater J 2020 39 05 703 720 32213767 10.4012/dmj.2019-288

[11] Loushine B A Bryan T E Looney S W Setting properties and cytotoxicity evaluation of a premixed bioceramic root canal sealer J Endod 2011 37 05 673 677 21496669 10.1016/j.joen.2011.01.003

[12] Rodríguez-Lozano F J García-Bernal D Oñate-Sánchez R E Ortolani-Seltenerich P S Forner L Moraleda J M Evaluation of cytocompatibility of calcium silicate-based endodontic sealers and their effects on the biological responses of mesenchymal dental stem cells Int Endod J 2017 50 01 67 76 26660310 10.1111/iej.12596

[13] Jung S Sielker S Hanisch M R Libricht V Schäfer E Dammaschke T Cytotoxic effects of four different root canal sealers on human osteoblasts PLoS One 2018 13 03 e0194467 29579090 10.1371/journal.pone.0194467 PMC5868789

[14] López-García S Pecci-Lloret M R Guerrero-Gironés J Comparative cytocompatibility and mineralization potential of Bio-C sealer and totalfill BC sealer Materials (Basel) 2019 12 19 3087 31546696 10.3390/ma12193087 PMC6804055

[15] Lim M Jung C Shin D-H Cho Y B Song M Calcium silicate-based root canal sealers: a literature review Restor Dent Endod 2020 45 03 e35 32839716 10.5395/rde.2020.45.e35 PMC7431927

[16] Park M G Kim I R Kim H J Kwak S W Kim H C Physicochemical properties and cytocompatibility of newly developed calcium silicate-based sealers Aust Endod J 2021 47 03 512 519 33894082 10.1111/aej.12515

[17] Agrafioti A Koursoumis A D Kontakiotis E G Re-establishing apical patency after obturation with Gutta-percha and two novel calcium silicate-based sealers Eur J Dent 2015 9 04 457 461 26929681 10.4103/1305-7456.172625 PMC4745224

[18] Aminoshariae A Kulild J C The impact of sealer extrusion on endodontic outcome: a systematic review with meta-analysis Aust Endod J 2020 46 01 123 129 31449355 10.1111/aej.12370

[19] Kim J H Cho S Y Choi Y Kim D H Shin S J Jung I Y Clinical efficacy of sealer-based obturation using calcium silicate sealers: a randomized clinical trial J Endod 2022 48 02 144 151 34856212 10.1016/j.joen.2021.11.011

[20] Cohen B I Pagnillo M K Musikant B L Deutsch A S Formaldehyde evaluation from endodontic materials Oral Health 1998 88 12 37 39 10323136

[21] Leonardo M R Bezerra da Silva L A Filho M T Santana da Silva R Release of formaldehyde by 4 endodontic sealers Oral Surg Oral Med Oral Pathol Oral Radiol Endod 1999 88 02 221 225 10468467 10.1016/s1079-2104(99)70119-8

[22] Cohen B I Pagnillo M K Musikant B L Deutsch A S An in vitro study of the cytotoxicity of two root canal sealers J Endod 2000 26 04 228 229 11199724 10.1097/00004770-200004000-00008

[23] Ho Y C Huang F M Chang Y C Cytotoxicity of formaldehyde on human osteoblastic cells is related to intracellular glutathione levels J Biomed Mater Res B Appl Biomater 2007 83 02 340 344 17385229 10.1002/jbm.b.30801

[24] Merdad K Pascon A E Kulkarni G Santerre P Friedman S Short-term cytotoxicity assessment of components of the epiphany resin-percha obturating system by indirect and direct contact millipore filter assays J Endod 2007 33 01 24 27 17185123 10.1016/j.joen.2006.10.003

[25] Schweikl H Schmalz G Federlin M Mutagenicity of the root canal sealer AHPlus in the Ames test Clin Oral Investig 1998 2 03 125 129 10.1007/s007840050057 9927913

[26] Ciasca M Aminoshariae A Jin G Montagnese T Mickel A A comparison of the cytotoxicity and proinflammatory cytokine production of EndoSequence root repair material and ProRoot mineral trioxide aggregate in human osteoblast cell culture using reverse-transcriptase polymerase chain reaction J Endod 2012 38 04 486 489 22414834 10.1016/j.joen.2011.12.004

[27] Sousa C JA Montes C RM Pascon E A Loyola A M Versiani M A Comparison of the intraosseous biocompatibility of AH Plus, EndoREZ, and Epiphany root canal sealers J Endod 2006 32 07 656 662 16793475 10.1016/j.joen.2005.12.003

[28] Huang F M Yang S F Chang Y C Effects of root canal sealers on alkaline phosphatase in human osteoblastic cells J Endod 2010 36 07 1230 1233 20630305 10.1016/j.joen.2010.03.001

[29] Kim T G Lee Y H Lee N H The antioxidant property of pachymic acid improves bone disturbance against AH plus-induced inflammation in MC-3T3 E1 cells J Endod 2013 39 04 461 466 23522537 10.1016/j.joen.2012.11.022

[30] Beck G R Jr. Sullivan E C Moran E Zerler B Relationship between alkaline phosphatase levels, osteopontin expression, and mineralization in differentiating MC3T3-E1 osteoblasts J Cell Biochem 1998 68 02 269 280 9443082 10.1002/(sici)1097-4644(19980201)68:2<269::aid-jcb13>3.0.co;2-a

[31] Borges R P Sousa-Neto M D Versiani M A Changes in the surface of four calcium silicate-containing endodontic materials and an epoxy resin-based sealer after a solubility test Int Endod J 2012 45 05 419 428 22150403 10.1111/j.1365-2591.2011.01992.x

[32] Lee B N Hong J U Kim S M Anti-inflammatory and osteogenic effects of calcium silicate-based root canal sealers J Endod 2019 45 01 73 78 30558800 10.1016/j.joen.2018.09.006

[33] Belal R S Edanami N Yoshiba K Comparison of calcium and hydroxyl ion release ability and in vivo apatite-forming ability of three bioceramic-containing root canal sealers Clin Oral Investig 2022 26 02 1443 1451 10.1007/s00784-021-04118-w 34398328

[34] Siqueira J F Jr. Lopes H P Mechanisms of antimicrobial activity of calcium hydroxide: a critical review Int Endod J 1999 32 05 361 369 10551109 10.1046/j.1365-2591.1999.00275.x

[35] Lopez-Cazaux S Bluteau G Magne D Lieubeau B Guicheux J Alliot-Licht B Culture medium modulates the behaviour of human dental pulp-derived cells: technical note Eur Cell Mater 2006 11 35 42, discussion 42 16485235 10.22203/ecm.v011a05

[36] Koutroulis A Kuehne S A Cooper P R Camilleri J The role of calcium ion release on biocompatibility and antimicrobial properties of hydraulic cements Sci Rep 2019 9 01 19019 31836731 10.1038/s41598-019-55288-3 PMC6910940

[37] Benetti F Queiroz ÍOA Cosme-Silva L Conti L C Oliveira S HP Cintra L TA Cytotoxicity, biocompatibility and biomineralization of a new ready-for-use bioceramic repair material Braz Dent J 2019 30 04 325 332 31340221 10.1590/0103-6440201902457

[38] Debelian G Trope M The use of premixed bioceramic materials in endodontics G Ital Endod 2016 30 70 80

[39] Penha da Silva P J Marceliano-Alves M F Provenzano J C Dellazari R LA Gonçalves L S Alves F RF Quality of root canal filling using a bioceramic sealer in oval canals: a three-dimensional analysis Eur J Dent 2021 15 03 475 480 33535249 10.1055/s-0040-1722095 PMC8382469

